# Sexism and Feminist Conspiracy Beliefs: Hostile Sexism Moderates the Link Between Feminist Conspiracy Beliefs and Rape Myth Acceptance

**DOI:** 10.1177/10778012241234892

**Published:** 2024-03-04

**Authors:** Daniel Jolley, Silvia Mari, Tanya Schrader, Darel Cookson

**Affiliations:** 1 6123 University of Nottingham, University Park, Nottingham NG7 2RD, UK; 2 9305 University of Milano-Bicocca, 20126 Milano, Italy; 3 7703 Staffordshire University, Stoke-on-Trent ST4 2DF, UK; 4 6122 Nottingham Trent University, Nottingham NG1 4FQ, UK

**Keywords:** feminist conspiracy beliefs, hostile sexism, sexist ideology, prejudice, rape myth acceptance

## Abstract

This research examined the effect of belief in feminist conspiracy theories and sexist ideology on endorsing rape myths. Study 1 (*N* = 201) uncovered that the relationship between feminist conspiracy beliefs and rape myth acceptance was conditional on higher levels of hostile sexism. Study 2 (*N* = 552) demonstrated that for those with higher hostile sexism, exposure to feminist conspiracy theories (vs. control) increased feminist conspiracy beliefs, which were then associated with rape myths. The current research suggests that the link between feminist conspiracy beliefs and rape myths could result from such beliefs upholding a hostile sexist view of women.

## Introduction

*Conspiracy theories* are commonly defined as explanations for events that involve secretive and (perceived) powerful groups covering up information to suit their interests (Douglas et al., 2019). Such theories can implicate a range of different groups, from scientists (e.g., climate change is a hoax because scientists fake their data to make a profit, Jolley & Douglas, 2014) to people from minority groups (e.g., that Jewish people are involved in significant events such as 9/11; Jolley et al., 2020). Conspiracy beliefs are persuasive; many millions believe in conspiracy theories (e.g., Douglas et al., 2019). Considering the popularity of conspiracy beliefs, understanding how conspiracy beliefs may impact the smooth running of societies is essential. In the current research, we focused on the impact of intergroup conspiracy beliefs. Specifically, we explored the relationship between belief in feminist conspiracy theories that propose feminists are involved in secret malevolent plots and schemes aimed at destabilizing gender hierarchy at the expense of men and sexist ideology on sexual prejudice held toward women—the endorsement of rape myths.

### The Psychology of Conspiracy Theories

Scholars have made great strides in understanding why people subscribe to conspiracy theories (see Douglas et al., 2019, for an interdisciplinary review). It is widely understood that conspiracy beliefs bloom around periods of uncertainty and anxiety in society, where such needs are often provoked by moments of crisis (van Prooijen & Douglas, 2017). However, while conspiracy theories may seem appealing, they are not psychologically satisfying (Douglas et al., 2019; Jolley et al., 2022). Research has also demonstrated that conspiracy beliefs can have attitudinal (e.g., racist attitudes, Jolley et al., 2020) and behavioral consequences (e.g., reduced intention to vaccinate a (fictional) child, Jolley & Douglas, 2014; see Jolley et al., 2022 for a review).

#### Intergroup Conspiracy Theories

Conspiracy theorizing can also take an intergroup focus, where minority groups with varied social power (e.g., Jews, Muslims, immigrants, feminists) are the target of conspiracy theories. Such a focus means people often subscribe to conspiracy theories that target another social group to their own (see Douglas et al., 2019). For example, in politics, people are more likely to believe that the political opposition is involved in secret plots and schemes than their own political party (e.g., voter fraud; Claassen & Ensley, 2016). This example underscores the importance of motivated reasoning when considering why people subscribe to conspiracy beliefs. Since people likely filter events through the lens of their political ideologies, conspiracy theories that uphold ideological systems are likely particularly appealing—such as the idea that *other* political groups conspire (see Douglas et al., 2019). However, with such an intergroup element, conspiracy beliefs can interfere with positive intergroup relations (see Jolley et al., 2020). For example, endorsement of Jewish conspiracy theories is correlated with prejudice and discrimination toward Jewish people (e.g., Bilewicz et al., 2013). Extending this work, Jolley et al. (2020), in an experimental design, demonstrated how exposure to Jewish conspiracy theories can increase prejudice, which can even generalize to other groups not involved in the alleged conspiracies. It is plausible that conspiracy theories concerning feminists may also have attitudinal and behavioral consequences.

### Feminist Conspiracy Beliefs and (Sexual) Prejudice

The feminist movement, because it aims to challenge the status quo (e.g., challenging traditional gender roles or lobbying for rape reform), has prompted conspiracy theories. Such conspiracy theories claim feminists secretly plot something sinister behind the scenes against men to support their self-interests (see Kempker, 2018). Some media studies have described how misandry has been used in men's rights communities (the manosphere) and infiltrated more mainstream contexts (Marwick & Caplan, 2018). The term refers to a misogynistic and conspiratorial view of feminism, which Marwick and Caplan (2018) argue privileges women's rights over men's and portrays it as a man-hating movement that victimizes men (see also Hopton & Langer, 2022). In this way, feminist conspiracy theories are a specific set of beliefs that go further than the mere stereotype of feminists. The latter may include negative beliefs of women hating men alongside positive beliefs (such as women being knowledgeable), whereas conspiracy beliefs are routed in a *conspiracy*. Feminist conspiracy theories are surprisingly prevalent. Eighteen percent of Spanish respondents in a survey support the conspiracy theory that feminists want to secretly dismantle the traditional family (Gualda et al., 2019). In contrast, others argue that feminists are conspirators in league with a secret world government (see Kempker, 2018).

A similar phenomenon is gender conspiracy beliefs, which propose gender studies were introduced secretly against the Catholic Church to harm traditional values and social arrangements (Marchlewska et al., 2019). As argued by the authors, “gender conspiracy beliefs might be an important factor predicting sexual prejudice” (p. 771). Gender conspiracy theories implicitly involve a broad range of groups alleged to be involved in secret plots and schemes (e.g., gender-equality activists, feminists, and the LGBTQ+ community). In contrast, feminist conspiracy theories focus on a specific group. While we expect gender and feminist conspiracy beliefs to be associated, it is plausible that feminist conspiracy theories could influence *prejudices toward women* more than gender conspiracy beliefs.

Specifically, a feminist is often prototypically female (vs. male)—as illuminated by a Young Women's Trust, 2019 poll, which, with a sample of 18–30-year-old participants, uncovered that 57% of women versus 33% of men identified as being a “feminist.” Therefore, prejudices held toward “feminists” could generalize to subtypes of women such as “housewives” and “career women,” but also “women” more broadly (stereotype content model; Fiske et al., 2002). This is akin to attitude generalization, where attitudes toward objects can generalize to other similar objects (e.g., Ratliff & Nosek, 2011). Attitude generalization can also include prejudices, such as Jewish conspiracy theories increasing prejudice against other, uninvolved groups in an experimental study (Jolley et al., 2020). Belief in feminist conspiracy theories may broadly increase prejudices against “women” and not be reserved solely for feminists because the conspiracy targets a subgroup of women. One negative attitude (toward feminists) provoked by a conspiracy theory generalizes to another group (women).

One key example of prejudice toward women is rape myths, which refer to prejudiced or stereotypical attitudes regarding rape, whereby violence against women is justified (Burt, 1980; Lonsway & Fitzgerald, 1994). Specifically, rape myths blame the rape victim for their assault, and the blame for the perpetrator is absolved (Payne et al., 1999). Common myths cited included “*women enjoy being raped*” and that women who are dressed in a certain way are “*asking for it*” (Maxwell & Scott, 2014). Understanding the psychological contributors to upholding rape myths is an important endeavor. Progress has been made, with research uncovering that the perception of violating gender roles is a strong correlate of rape myths (Davies et al., 2012). Pertinently, people who believe that feminists are conspiring against men and do not conform to gender roles are also likely to hold sexual prejudices. Indeed, as discussed earlier, intergroup conspiracy beliefs have been shown to breed prejudice toward the target of the conspiracy alongside other uninvolved groups through attitude generalization (e.g., Jolley et al., 2020). Therefore, the belief that feminists are conspiring may also be positively associated with endorsing rape myths. We sought to explore this possibility.

### Feminist Conspiracy Beliefs, Sexist Ideology, and Rape Myths

Endorsing the belief that feminists are acting secretly for their self-interests (e.g., privileging women's rights over men's or secretly dismantling the family) may be particularly appealing if such a belief supports an individual's political ideology. As Douglas et al. (2019) discussed, people likely evaluate events and circumstances via their political predispositions. While research has typically focused on political orientation, we propose that sexist ideologies likely also play a role in conspiracy beliefs. Specifically, feminist conspiracy beliefs could appeal to individuals with heightened sexist attitudes toward women because such conspiracy beliefs uphold a sexist ideological worldview.

Glick and Fiske (1996) posited that sexist ideology about women could comprise two correlated but oppositely valenced attitudes: hostile and benevolent sexism. Hostile sexism paints women in a negative light. Women are categorized as deceitful or too easily offended, which involves overt antipathy toward women who may threaten the gender hierarchy, like feminists. On the other hand, benevolent sexism could be seen as apparently positive. Women are posed as fragile and thus should be protected by men, to name an example; however, benevolent sexism reinforces women's subordinate roles. Although they work together, benevolent sexism rewards women who embrace being “traditionally” female, while hostile sexism penalizes women who deviate from the female stereotype (Connor & Fiske, 2018). Ramos et al. (2018) argue that both hostile and benevolent sexism indicate that women are, and should be seen as, less competent than men. Ambivalent sexism forms a complementary ideological system that justifies and maintains gender inequality. Feminist conspiracy beliefs could appeal to those who hold such an ideological system.

Moreover, such a sexist ideology could explain the link between feminist conspiracy beliefs and sexual prejudice. Women who violate traditional gender roles are likely to be prejudiced against by people who hold a stronger sexist ideology as a route to uphold gender inequality. Indeed, research has found that hostile sexism—but not benevolent—is positively predictive of rape myths (e.g., Glick & Fiske, 1997). However, other studies have found, depending on the circumstance, hostile and benevolent sexism are both associated (see also Angelone et al., 2021, who explored the subscales of benevolent sexism). Specifically, Abrams et al. (2003) argue that people who score more highly on benevolent sexism are likely to blame victims *when* the victim is seen as not maintaining “ladylike” standards following acquaintance—but not a stranger—rape. In a meta-analysis by Suarez and Gadalla (2010), where hostile and benevolent were combined, a positive association was demonstrated with endorsing rape myths. Therefore, feminist conspiracy beliefs and rape myths could be linked because such a connection perfectly upholds a sexist ideological system (i.e., feminist conspiracy beliefs inspiring prejudice encapsulates animosity toward women). We also sought to examine this possibility.

## Current Research

Two studies investigated the interaction between ambivalent sexism and belief in feminist conspiracy theories on endorsing rape myths. Study 1 tested whether a belief in a feminist conspiracy was a unique predictor of rape myth acceptance while controlling for other known predictors. We also examined whether this relationship between feminist conspiracy theories and rape myths was *conditional* on heightened sexist attitudes. In Study 2, we experimentally manipulated exposure to feminist conspiracy theories (vs. control). We examined whether those with sexist attitudes were more likely to endorse a feminist conspiracy after exposure, which would be associated with increased acceptance of rape myths. All materials and data can be viewed at https://osf.io/qamxk.

## Study 1

Study 1 explored the links between feminist conspiracy beliefs, ambivalent sexism, and the association with rape myth acceptance. Participants were asked to indicate their belief in conspiracy theories—in both a measure of general notions of conspiracy (Brotherton et al., 2013) and a specific belief in a feminist conspiracy. By including the general measure of conspiracy theorizing, we could control for the general tendency to engage in conspiracy theorizing and thus showcase the unique power of feminist conspiracy beliefs. Alongside conspiracy beliefs, existing predictors of rape myth acceptance were completed by participants, enabling us to determine the unique predictive power of feminist conspiracy beliefs. These include a measure of ambivalent sexism (hostile and benevolent), our moderator variables, and a measure of belief in a just world. Lerner (1997) defines beliefs in a just world as a construct whereby people have faith that the world is a fair place and everyone receives what they deserve. This provides reassurance that if good people take the required precautions, nothing bad will occur (Lerner, 1997). Research has found support that relationships between just world beliefs and rape myths exist (e.g., Lonsway & Fitzgerald, 1994). Also, identifying as a feminist has been associated with lower rape myths (Kunst et al., 2019), which we measured. We hypothesized that alongside the existing predictors, belief in a feminist conspiracy would be a significant unique predictor of accepting rape myths when controlling for such factors (*H*1). We also hypothesized an interaction between ambivalent sexism and belief in feminist conspiracy theories, where the link between conspiracy beliefs and rape myths would be strengthened for those with high levels of ambivalent sexism (*H*2).

### Method

#### Participants and Design

Two hundred and one participants (53 male, 146 female, and two who would rather not say, *M_age_ *= 26.95, *SD* = 10.77) were recruited via snowball sampling using social media and poster advertisements. All participants were residents of the United Kingdom and received no compensation. A correlational design was employed. Feminist conspiracy beliefs, ambivalent sexism (hostile and benevolent), and rape myth acceptance were measured alongside control variables of general conspiracy beliefs, just world beliefs, gender identity, age, political orientation, and feminist identification. A small to medium-sized effect size (*R*^2^ = .08) for a nine-predictor variable regression (testing *H*1) required a minimum sample size of approximately 189 participants for 80% power of detecting the effect using GPower.

#### Materials and Procedure

Unless stated, participants completed their agreement on a 7-point scale (1 = *strongly disagree*, 7 = *strongly agree*).

To begin, participants first provided their informed consent. Participants were then asked to complete an item that measured belief in a feminist conspiracy (“Feminists are involved in secret plots and schemes”), which, while only one item, does have high face validity, followed by the Conspiracist Ideation Scale (Brotherton et al., 2013), which measures general notions of conspiracy belief that include 15 items (e.g., “The government manipulate, fabricate, or suppress evidence in order to deceive the public.” α = .95). These two measures were counterbalanced.

Next, a measure of sexism was completed using the Ambivalent Sexism Inventory (Glick & Fiske, 1996), which included two 11-item subscales: benevolent sexism (e.g., “Women should be cherished and protected by men.” α = .80) and hostile sexism (e.g., “Women seek to gain power by getting control over men.” α = .90). These scales were also counterbalanced. This was followed by the scale measuring beliefs in a just world (Lerner, 1997) comprising of an eight-item scale (e.g., “I feel that people generally earn the rewards and punishments that they get in this world.” α = .90).

Finally, participants completed a measure of rape myth acceptance focused on women (Updated Illinois Rape Myth Scale, McMahon & Farmer, 2011). The scale included 22 items (e.g., “If a woman is raped while she is drunk, she is at least somewhat responsible for letting things get out of hand.” α = .91). After completing all measures, participants were asked to complete demographic questions that also included a question on whether they identified as a feminist (“How would you rate your personal degree of feminism?”) on a scale of 1 (s*trong antifeminist*) to 7 (s*trong feminist*), how they self-identified their gender (1 = *male*, 2 = *female*, 3 = *trans*, 4 =* [open text]*, 5 = *rather not say*), and their political orientation (1 [*very left wing*] to 7 [*very right wing*]). Participants were then debriefed and thanked.

### Results and Discussion

Descriptive statistics and correlations between all variables are presented in Table 1. As expected, belief in feminist conspiracy theories was significantly positively correlated with rape myth acceptance, alongside hostile and benevolent sexism and belief in general conspiracy theorizing. Belief in general conspiracy theorizing was also positively correlated with the same variables as feminist conspiracy beliefs. Older participants and those identifying less as feminists were more likely to subscribe to feminist conspiracy theories and score higher on general conspiracy theorizing. Right-wing participants were more likely to indicate a higher belief in feminist conspiracy theories. Older participants, more right-wing participants, and those scoring lower on feminist identity were more likely to endorse rape myths. Further, as expected, males (vs. females) scored significantly lower on feminist identity but significantly higher on benevolent sexism and rape myth acceptance; males were also marginally significantly more likely to score higher on belief in a feminist conspiracy and hostile sexism than females (see Supplementary Materials Table S1 for statistics).

**Table 1.** Means and Pearson Product-Moment Correlations for All Variables in Study 1 (*N* = 201).

		*M* (*SD*)	1	2	3	4	5	6	7	8	9
1	Age	26.95 (*10.76*)	–	−.07	−.07	.27***	.20**	.04	.01	−.13	.19**
2	Feminist identity	4.68 (*1.41*)		–	−.34***	−.14*	−.38***	−.24**	−.60***	−.09	−.30***
3	Political orientation	3.27 (*1.20*)			–	−.02	.27***	.21**	.34***	.05	.17*
4	General conspiracy theorizing	3.84 (*1.36*)				–	.38***	.22**	.21**	−.11	.15*
5	Feminist conspiracy	2.25 (*1.26*)					–	.28***	.51***	.02	.41***
6	Benevolent sexism	3.00 (*0.98*)						–	.41***	.20**	.46***
7	Hostile sexism	2.82 (*1.14*)							–	.25***	.54***
8	Just world beliefs	3.61 (*1.17*)								–	.16*
9	Rape myth acceptance	1.85 (*0.76*)									–

* *p* < .05, ** *p* < .01, *** *p* < .001.

To produce a robust test of our prediction that feminist conspiracy theories uniquely predict rape myth acceptance (incremental validity), a four-step hierarchal, multiple linear regression was undertaken with rape myth acceptance as the criterion (see Table 2). In Step 1, background variables of age, gender (males vs. females), politics, and feminist identification were included as demographic controls because each variable correlated with the dependent variable (see Table 1). In Step 2, ambivalent sexism (benevolent and hostile) and belief in a just world were included, as each variable predicts rape myths. In Step 3, we then entered general conspiracy theorizing, followed by belief in a feminist conspiracy in Step 4.

**Table 2.** Hierarchical Regression Analysis Predicting Rape Myth Acceptance Using Gender, Age, Political Orientation, Feminist Identification, Benevolent Sexism, Hostile Sexism, Belief in a Just World, General Notions, and a Feminist Conspiracy Belief (Study 1, *n* = 199).

		Step 1	Step 2	Step 3	Step 4
1	Gender (0 = male, 1 = female)	−.18**	−.11*^¥^*	−.10*^¥^*	−.10
1	Age	.14*	.16**	.17**	.15*
1	Political orientation	.12	.00	−.00	−.03
1	Feminist identification	−.22***	.06	.06	.07
2	Benevolent sexism	–	.26***	.26***	.26***
2	Hostile sexism	–	.44***	.45***	.39***
2	Belief in a just world	–	.02	.02	.03
3	General conspiracy theorizing	–	–	−.04	−.08
4	Feminist conspiracy	–	–	–	.17*
*R*²		.16	.39	.39	.41
*R*² change		–	.23***	.00	.02*

*^¥^* *p* < .10, * *p < .*05, ** *p *< .01, *** *p* < .001.

At Step 1, age, feminist identity, and gender were significant predictors; specifically, men (vs. women), older participants, and those who scored lower on feminist identification were more likely to accept rape myths, while political orientation was nonsignificant. At Step 2, age remained a significant predictor, alongside benevolent and hostile sexism. Gender was a marginally significant predictor, and political orientation, feminist identification, and belief in a just world were nonsignificant. Adding general conspiracy beliefs in Step 3 did not change the model and was a nonsignificant predictor. At Step 4, feminist conspiracy beliefs significantly improved the model fit, where belief in a feminist conspiracy theory was a significant positive predictor, alongside age, benevolent, and hostile sexism. Gender, political orientation, feminist identification, belief in a just world, and general conspiracy theorizing were nonsignificant. Therefore, belief in feminist conspiracy theories *uniquely* predicted the acceptance of rape myths alongside other known factors, supporting our first hypothesis.

Next, moderation analysis was run with PROCESS Model 1 using 5,000 bootstrapped samples (Hayes, 2015) to examine the moderating effect of ambivalent sexist ideology (hostile and benevolent, respectively) on the relationship between feminist conspiracy theories and rape myth acceptance. Each level of the moderator was generated by the pick-a-point method (Hayes, 2013): low (standardized variable −1 *SD*), moderate (standardized variable: 0), and high (standardized variable: +1 *SD*)*.* Because the demographic variables (age, gender, political orientation, and feminist identification) were correlated with rape myths, these factors were controlled for in the analyses. The moderation analysis showcased a significant interaction effect between hostile sexism and feminist conspiracy beliefs on rape myth acceptance (*b *= .08, *p* = .015, 95% CI [0.02–0.14], and accounted for 2% of the overall variance of rape myth acceptance, *F*(1, 191) = 6.07, *p* = .015).

A simple slope test revealed that when hostile sexism was higher, the effect of feminist conspiracy beliefs on rape myth acceptance was significant in a positive direction (*b* = .14, *p* = .002, 95% CI [0.05–0.24]). However, when hostile sexism was at a moderate and low level, the effect was nonsignificant (*b* = .06, *p* = .20, 95% CI [−0.03–0.14]; *β* = −.03, *p* = .641, 95% CI [−0.16–0.10], respectively). The Johnson–Neyman technique was also applied; it identifies regions in the range of the moderator variable where the effect of the focal predictor on the outcome is statistically significant (see Hayes & Matthes, 2009). The results showed that for participants with a score on the hostile sexism measure superior to or equal to the average +.34 *SD*, feminist conspiracy beliefs significantly predicted rape myths.

Benevolent sexism, however, was found not to act as a moderator between feminist conspiracy beliefs and rape myth acceptance (benevolent × feminist conspiracy beliefs, *b* = .01, *p* = .673, 95% CI [−0.05–0.08], *F*(1, 191) = 0.179, *p* = .673). Therefore, the results partly support our second hypothesis, where the relationship between feminist conspiracy beliefs and rape myth acceptance is conditional on higher levels of hostile sexist ideology. Unexpectedly, benevolent sexist ideology did not act as a moderator.

These results provide empirical support that belief in a feminist conspiracy plays a unique role in predicting the acceptance of rape myths when controlling for other known predictors, general conspiracy theorizing, and demographics (i.e., gender and feminist identification). Importantly, hostile sexism moderated this relationship: the link between feminist conspiracy beliefs and rape myth acceptance was conditional on heightened hostile sexist ideology. Therefore, the study provides evidence that the links between feminist conspiracy beliefs and rape myths (sexual prejudice) could likely result from an individual's predispositions (see Douglas et al., 2019).

However, we also found that while benevolent sexism was shown to be predictive of rape myths, no interaction between benevolent sexism and belief in feminist conspiracy occurred. Feminist conspiracy theories arguably evoke a negative stereotype of feminists (and women, by extension), which aligns with the negative sentiment of hostile sexism. On the other hand, benevolent sexism paints women in a subjectively favorable light which may help explain why benevolent sexism does not act as a moderator between feminist conspiracy theories and rape myths in general. A similar pattern has emerged in other research, where negative stereotypes about feminists were strongly tied to hostile (rather than benevolent) sexism (Robnett et al., 2012). Such authors pose that this effect occurs because hostile sexism pertains most directly to gender-role violations. Indeed, the interaction between benevolent sexism and feminist conspiracy theories may only become apparent when *specific* crime elements are emphasized in a rape scene promoting role violations (Abrams et al., 2003). For example, benevolent sexism may be a moderator when a victim is seen as failing to be “ladylike” and thus activates negative stereotypes.

While Study 1 has uncovered a link between feminist conspiracy theories and sexual prejudice (rape myth acceptance) that is conditional on hostile sexism, the study's correlational design is a limitation of the study. We cannot explore a causal link between exposure to feminist conspiracy theories and prejudice against women, specifically in individuals who hold high hostile sexist attitudes. For example, whether an individual who harbors high levels of hostile sexism would be more likely to subscribe to feminist conspiracy theories after conspiracy exposure and if this, in turn, would be associated with the endorsement of rape myths. In Study 2, we sought to test this possibility.

## Study 2

We employed an experimental design to explore whether those with strong hostile sexist attitudes tend to endorse feminist conspiracy theories and report rape myths after feminist conspiracy exposure. Participants first completed measures of hostile sexism before being exposed to feminist conspiracy theories or nothing (control condition) using a methodological approach already applied in previous research on conspiracy beliefs (e.g., Jolley et al., 2020). Participants then indicated their belief in feminist conspiracy theories and acceptance of rape myths. We predicted (preregistration: https://aspredicted.org/TH1_TZS) a conditional effect on rape myths. Specifically, exposure to conspiracy theories that argue feminists are involved in malevolent plots and schemes that seek to threaten men's dominant position will appeal more to people who already hold ideological and biased visions of women (i.e., hostile sexism). For these individuals, exposure is likely to reinforce feminist conspiracy beliefs, which will be associated with rape myths (*H*1).

### Method

#### Participants and Design

Five hundred and seventy-eight participants (282 female, 288 male, three nonbinary, and three who prefer not to say, *M_age_ *= 40.20, *SD* = 12.39) were recruited from *Prolific*. Participants were all residents of the United Kingdom and received a small fee in exchange for their participation. A timer was used to identify participants who had spent less than 30 s reading the conspiracy theory material. Participants who failed this screening measure were removed from the analyses (*n* = 24). Two participants did have substantial missing data, so they were omitted. The final sample used for data analysis was 552 (270 females, 276 males, three nonbinary, and three who prefer not to say, *M_age_* = 40.46, *SD* = 12.44). Participants were randomly assigned to a pro-conspiracy condition (*n* = 262) or a control condition (*n* = 290) in a between-subjects design. Based on the effect size derived from Study 1 of conspiracy × hostile sexism on rape myths (.02), G*Power recommended a minimum of 550 participants at 80% power for a moderation analysis (three predictors used in the linear multiple regression option). We aimed to recruit 578, expecting a 5% dropout rate.

#### Materials and Procedure

After participants provided their informed consent, participants were asked to complete a measure of hostile sexism (α = .93), as in Study 1. Participants were then exposed to information that supported conspiracy theories about feminists being involved in secret plots and schemes (pro-conspiracy condition) or where no information was given (control condition, akin to previous research, e.g., Jolley et al., 2020). Participants assigned to the pro-conspiracy condition were told that they would be asked to read a short excerpt from a recent internet article about feminists and that they would be asked some questions about the except later in the study. The pro-conspiracy article argued that feminists are actively working, in secret, to weaken traditional family life, that the #metoo movement was hijacked by feminists as a way to marginalize men to increase representation quotes for women, and that feminists are conspiring to remove free speech from men. An extract from the article was as follows:

There is also a significant amount of evidence showing that feminists are conspiring to remove free speech from men, as can be seen by increasing calls for misogyny to be adopted as a hate crime… …Many also argue that feminists are actively working, in secret, to weaken traditional family life…

Participants then indicated their belief in feminist conspiracy theories using 5-items (e.g., “Feminists are often involved in secret plots and schemes”; “Feminists are working in secret to weaken the traditional family for their own gain,” α = .94) on a scale of 1 (*strongly disagree*) to 7 (*strongly agree*), thus overcoming the issue of the single item used in Study 1. An Exploratory Factor Analysis confirmed that the items of belief in feminist conspiracy theories and hostile sexism load substantially on the predicted scale (see Supplementary Materials). Also, the success of the manipulation had been previously tested in a small-scale pilot study (*N* = 44, *M_age_* = 32.09 (*SD* = 9.71), 29 females, spent >30 reading the pro-conspiracy manipulation), where controlling for feminist identity, exposure to feminist conspiracy theories was shown to significantly increase feminist conspiracy beliefs (*M* = 2.85, *SD* = 1.81, *n* = 21) compared to a control condition (*M* = 2.07, *SD* = 0.82, *n* = 23, *F*(1, 43) = 5.18, *p* = .028, *η*^2^* *= .13).

Next, participants completed a measure of rape myth acceptance as used in Study 1 (α = .92). After completing all measures, participants were asked to complete demographic questions that also included a question on whether they identified as a feminist, as in Study 1. Time was then taken to debrief participants on the study aims and thank them for participating.

### Results and Discussion

#### Data Checking

First, we examined relationships between demographics and measured variables (see Supplementary Materials Table S2 for a full correlational table). Across experimental conditions, older participants were less likely to be feminists (*r* = −.09, *p* = .027) and more likely to accept rape myths (*r* = .13, *p* = .002). Participants who had a stronger feminist identification were, in general, less likely to be right wing (*r* = −.51, *p* < .001), believe in feminist conspiracy theories (*r* = −.58, *p* < .001), hold hostile sexist attitudes (*r* = −.60, *p* < .001), and accept rape myths (*r* = −.46, *p* < .001). More right-wing participants indicated higher feminist conspiracy beliefs (*r* = .44, *p* < .001), hostile sexist attitudes (*r* = .45, *p* < .001), and acceptance of rape myths (*r* = .41, *p* < .001). Males also reported significantly higher scores on variables than females (see Supplementary Materials Table S3 for descriptive and *t*-test statistics for all variables). Also, feminist conspiracy beliefs, hostile sexism, and rape myth acceptance were all positively correlated.

A one-way analysis of variance (ANOVA) was then conducted to examine the effect of exposure to feminist conspiracy theories on conspiracy beliefs. Results demonstrated that belief in feminist conspiracy theories was significantly higher in the conspiracy condition (*M* = 2.80, *SD* = 1.48) than in the control (*M* = 2.14, *SD* = 1.35), *F*(1, 546) = 36.035, *p* < .001, *η*^2^* *= .07. Following this, a further one-way ANOVA was conducted to determine the effects of exposure to feminist conspiracy theories on rape myths. There were no significant differences uncovered between conditions, *F*(1, 546) = 0.044, *p *= .834, *η*^2^* *= .00 (conspiracy condition *M* = 1.97 [*SD* = 0.78], control condition *M* = 1.93 [*SD* = .0.84]). Although there were no direct effects on the dependent measure, we predicted that the difference between conspiracy exposure (vs. control) on rape myths would be conditional on stronger sexist attitudes and increasing belief in feminist conspiracy theories. An examination of the interaction between conspiracy exposure and sexist attitudes was tested via a moderated mediation analysis.

#### Hypothesis Testing

**Figure 1.**
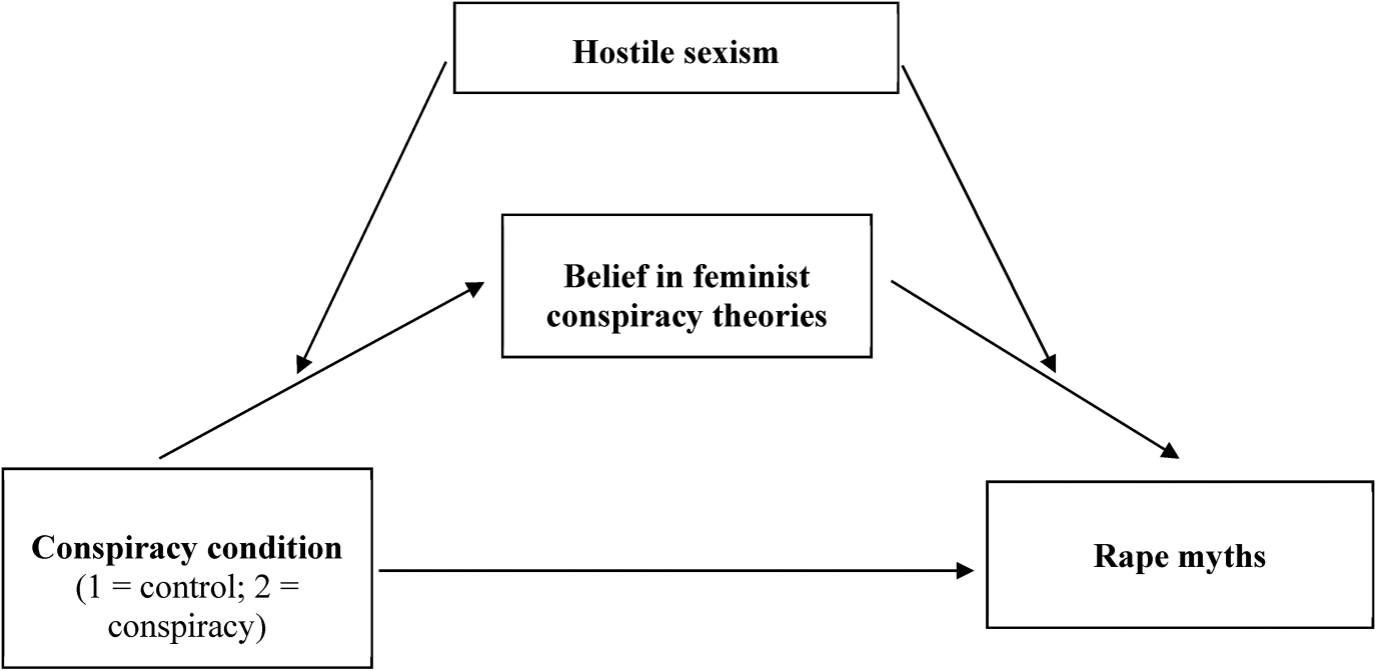
A path diagram to present the predicted moderated mediations (Model 58), including conspiracy exposure (predictor), belief in conspiracy theories (mediator), and rape myth acceptance (outcome), conditioned by hostile sexism (moderator) in Study 2.

A moderated mediation analysis was conducted to test our hypothesis exploring the interaction effect between belief in feminist conspiracy theories and hostile sexism on rape myths after conspiracy exposure (vs. control), using PROCESS macro for SPSS, Model 58 (Hayes, 2015). As shown in Figure 1, Model 58 is a saturated model including predictor (conspiracy exposure), mediator (conspiracy beliefs), and outcome (rape myths), in which we predict the effects of the predictor on the mediator and mediator on the outcome to be conditioned by an additional variable (hostile sexism). As in Study 1, each level of the moderator was generated by the pick-a-point method (Hayes, 2013): low (standardized variable −1 *SD*), moderate (standardized variable: 0), and high (standardized variable: +1 *SD*)*.* Since the indirect effect is a nonlinear function of the moderator (i.e., the outcome depends on two hostile sexism values), no index of moderation is provided (see Hayes, 2015). We tested this model using the 5,000 bootstrapped methods, controlling for age, gender, politics, and feminist identification.

Supporting our predictions (see also Table 3), the difference in feminist conspiracy beliefs between conspiracy exposure (vs. control) was moderated by hostile sexism (conspiracy exposure × hostile, *b* = .16, *p *= .025, 95% CI [0.0210–0.3086], *F*(1, 538) = 5.0687, *p* = .025, *Mhostile* = 2.93, *SD* = 1.04). A simple slope test revealed that at all levels of hostile sexism, the effect of exposure on feminist conspiracy beliefs was significant in a positive direction—however, as hostile sexism increased, the magnitude of the moderation strengthened (low: *b* = .54, *p* < .001, 95% CI [0.3331–0.7525], moderate: *b* = .72, *p* < .001, 95% CI [0.5671–0.8637], high: *b* = .89, *p* < .001, 95% CI [0.6749–1.1011]).

**Table 3.** Moderated Mediation Model of Hostile Sexism (Moderator), Conspiracy Exposure (Predictor), and Feminist Conspiracy Beliefs (Mediator, M) Predicting Rape Myths (Outcome) in Model 58 of PROCESS Macro (Study 2, *n* = 546).

	Feminist conspiracy beliefs (M)		Rape myths (Y)	
Variable	Coeff. (*SE*)	95% CI	Coeff. (*SE*)	95% CI
Condition	.72 (.08)***	**0.5671–0.8637**	.01 (.05)	−0.1000 to 0.112
Feminist conspiracy	–	–	**.08** (.03)*	**0.0256–0.1425**
Hostile sexism	.66 (.11)***	**0.4365–0.8778**	**.40** (.04)***	**0.3169–0.4738**
Condition × hostile sexism	.16 (.07)*	**0.0210–0.3086**	–	–
Feminist conspiracy × hostile sexism	–	–	***.03 (.01)^***	***−0.0039 to 0.0591***
Age	−.00 (.01)	−0.0063 to 0.0058	**.01** (.00)*	**0.017–0.0097**
Feminist identity	−.16 (04)***	**−0.2390 to −0.841**	.00 (.03)	−0.0503 to −0.0522
Gender (0 = male, 1 = female)	.07 (.08)	−0.0770 to 0.2318	−.06 (.05)	−0.1617 to 0.0396
Political orientation	.07 (.03)*	**0.0117–0.1391**	**.05** (.02)*	**0.0094–0.0934**
Constant	−.68 (.31)***	**−1.2926 to −0.864**	**1.60** (.21)***	**1.1921–2.0053**
	*R*^2^ = .64 *F*(7, 538) = 138.0161, *p* < .001		*R*^2^ = .52 *F*(8, 537) = 72.505, *p* < .001	

*Note.* A **bold** typeface indicates a significant effect ***. Bold italics*** typeface indicates a marginally significant effect.

*^* *p *< .10, * *p <* .05, *** *p* < .001.

Hostile sexism was also found to marginally moderate the link between belief in feminist conspiracy theories and rape myths (feminist conspiracy beliefs × hostile), *b* = .33, *p *= .085, 95% CI [−0.0039–0.0591], *F*(1, 537) = 2.97, *p* = .085). A simple slope test revealed that at the high (*b* = 0.11, *p* < .001, 95% CI [0.0566–0.1694]) and moderate (*b* = .08, *p* = .001, 95% CI [0.0256–0.1425]) levels of hostile sexism there was a significant difference in the positive direction, but those at the lower (*b *= .06, *p* = .16, 95% CI [−0.0212–0.1315]) values were nonsignificant. As in Study 1, the Johnson–Neyman technique was applied. The results showed that, for participants with a score on the hostile sexism measure superior to or equal to the average + −.60 *SD*, feminist conspiracy beliefs significantly increased rape myths.

We then examined whether an indirect effect of conspiracy exposure on rape myths through belief in feminist conspiracy theories would vary with hostile sexism. Results demonstrated a significant positive indirect effect for high and moderate hostile sexism (indirect effect .10, 95% CI [0.0321–0.1815]; indirect effect .06, 95% CI [0.0131–0.1114], respectively). The lower value was nonsignificant (indirect effect .03, 95% CI [−0.0185–0.0812]. As expected, this finding demonstrates that an increase in feminist conspiracy beliefs after conspiracy exposure (vs. control) being associated with rape myths (indirect effect) is conditional on higher levels of hostile sexism (moderator).

In summary, Study 2 has extended the findings of Study 1 by showcasing the conditional effects of hostile sexism after conspiracy exposure: higher levels of hostile sexism were associated with a heightened belief in feminist conspiracy theories after exposure to conspiracy theories. In turn, feminist conspiracy beliefs were then associated with rape myths, but only for those with higher levels of hostile sexism. This work demonstrates that the link between feminist conspiracy beliefs and rape myths is likely due to the upholding of hostile sexist ideologies.

## General Discussion

This current research established that belief in feminist conspiracy theories predicts sexual prejudices toward women (rape myths), which was conditional on higher levels of hostile sexism. In Study 1, belief in a feminist conspiracy predicted rape myth acceptance when controlling for other known psychological predictors (i.e., ambivalent sexism and belief in a just world) alongside general conspiracy theorizing and relevant demographic variables. This effect was conditional on stronger hostile sexism. Study 2 extended these findings by demonstrating exposure to conspiracy theories (vs. control) increased belief in feminist conspiracy theories, which was associated with rape myths. Critically and supporting our predictions, the indirect effect was conditional on those with higher levels of hostile sexism. These findings provide novel evidence of the interactional effects of hostile sexism and feminist conspiracy theories that may negatively impact women.

These findings advance previous work in several ways. Here, we provide evidence that belief in, and exposure to feminist conspiracy theories, where feminists are depicted as aiming at destabilizing the power hierarchy based on gender purposely at the expense of men (which goes beyond a simple feminist stereotype), could increase rape myths acceptance. Notably, conspiracy beliefs about one group (feminists) elicited prejudice toward another group (women), further supporting attitude generalization (e.g., Jolley et al., 2020). Intergroup conspiracy theories may have wide-ranging consequences on intergroup relations (e.g., Bilewicz et al., 2013), which our work demonstrates can include feminist conspiracy beliefs inspiring prejudice. Moreover, our work demonstrating the impact of conspiracy beliefs on rape myths extends our understanding of the antecedents of rape myth acceptance. Rape myths can have a significant impact on legal issues. For example, Krahé et al. (2008) found that participants who endorsed rape myths were likelier to blame the victim and recommend shorter sentences for the defendant. Focusing on the social aspects of rape myths, longitudinal research has uncovered a link between rape proclivity and rape myth acceptance (e.g., O'Connor, 2021), with Egan and Wilson (2012) also uncovering rape myth acceptance among rape survivors predicting the probability of them reporting rape to the police. Thus, belief in feminist conspiracy theories may play a unique role in upholding myths about rape victims, further cementing the negative impact of rape myths.

Moreover, an overlooked area in the literature has been exploring the interaction effect of ideology and belief in conspiracy theories (see Douglas et al., 2019). Douglas et al. (2019) propose that conspiracy theories must align with an individual's predispositions (e.g., political ideology). Here, we offer, the first to our knowledge, evidence of motivated cognition with an interaction between ambivalent sexist ideology and belief in feminist conspiracy theories. In Study 1, the effect between belief in a feminist conspiracy and rape myth acceptance was conditional on higher levels of hostile sexism. This effect was replicated using an experimental design. For those with higher levels of hostile sexism, exposure to feminist conspiracy theories increased feminist conspiracy beliefs, which was then associated with increased rape myth acceptance. Our work demonstrates that exposure to conspiracy theories may have detrimental societal consequences on gender prejudices and that these consequences after exposure could be contingent on previously held ideologies.

Although there were no direct effects of conspiracy exposure on rape myths, we believe this does not undermine the findings (see also Hayes & Rockwood, 2017). We predicted exposure to feminist conspiracy theories would be conditional on sexist ideology. Those who scored higher on hostile sexism were likely to endorse feminist conspiracy theories, which would be associated with prejudicial attitudes. Our findings supported this prediction. Specifically, we found that exposure to conspiracy theories about feminists does not merely increase prejudice. Instead, a hostile sexist predisposition strengthened exposure, increasing conspiracy beliefs, which were then associated with rape myths. Thus, conspiracy theories that focus on feminists, when threatening the dominant position of men as in the manipulation text used in Study 2, seem to reinforce the previously held ideological and biased vision of women, thus exacerbating the animosity toward women in general and independently of the participant gender. Our results provide further evidence that conspiracy beliefs could be a symptom of upholding ideologies. However, a future research endeavor could also explore whether repeated exposure to feminist conspiracy theories could have a reverse effect on sexist attitudes; that is, exploring the possibility of whether prolonged conspiracy exposure could *foster* a hostile sexist ideological system.

However, our effects did appear to be restricted to hostile sexism. In Study 1, we found that benevolent sexism did not act as a moderator between feminist belief in conspiracy theories and prejudice. Hostile and benevolent are different constructs, where hostile sexist attitudes may align closer with conspiracy beliefs about feminists than benevolent sexism. Similarly, when focusing on negative stereotypes of feminists, hostile sexism was a stronger predictor than benevolent sexism (Robnett et al., 2012). Indeed, hostile sexism paints women negatively, where women are seen to be deceitful. In contrast, benevolent sexist attitudes perceive women in a much more (subjectively) favorable light (e.g., Connor & Fiske, 2018). A scenario highlighting that a woman is not acting as “ladylike” may inspire conspiracy beliefs to flourish for those with benevolent sexist attitudes.

Our work has some limitations that could also be addressed in future research. While we uncover links between feminist conspiracy beliefs and prejudicial attitudes, alongside illuminating *when* this relationship is pronounced (i.e., under heightened hostile sexism), our data do not provide evidence on *why* the link between feminist conspiracy beliefs and prejudice may exist. As with sexist ideology, it is plausible that feminist conspiracy theories could promote the perception that feminists (and women broadly) violate gender roles, which strongly correlates with prejudicial attitudes (e.g., Davies et al., 2012). Understanding *why* (e.g., gender-role violation) alongside *when* (i.e., heightened hostile sexism) conspiracy beliefs are linked with prejudice would be an essential next step in the research. Further, although we uncovered statistically robust evidence of the link between feminist conspiracy beliefs and sexual prejudice, even when controlling for hostile sexism, the effect size was small for the unique role of feminist conspiracy beliefs. Nonetheless, our research showcases how feminist conspiracy beliefs can still contribute to upholding problematic attitudes toward women.

A further limitation was that demand characteristics could have impacted the conspiracy exposure manipulation in Study 2. While participants were not told the research concerned feminist *conspiracies*, those in the conspiracy condition may have become aware of the true aims of the manipulation. In future research, a longitudinal design could provide further robust evidence of our hypotheses to address this issue. Moreover, previous work has shown how people derogate (perceived) deviant ingroup members (Marques & Paez, 1994). Therefore, future research could build on this prior work and explicitly explore how gender and feminist identification interact with feminist conspiracy beliefs and sexist ideology (e.g., female nonfeminists degrading female feminists). Future research should also explore prejudices directed toward other minority groups (e.g., trans/nonbinary individuals). Addressing bias toward gender minorities is a burgeoning area of research. Finally, our sample consisted solely of UK participants, but we know that culture can play a role in conspiracy beliefs. For example, Hornsey and Pearson (2022) have argued how conspiracy beliefs are higher in nations that are more corrupt, more collectivist, and lower in GDP per capita. Thus, considering the role of culture in strengthening or weakening the link between conspiracy beliefs, hostile sexism, and prejudices could be another crucial future research direction.

## Conclusion

In summary, the current research has further showcased the dangers of intergroup conspiracy theories. Specifically, in Study 1, we uncovered a unique link between belief in a feminist conspiracy and the endorsement of rape myths. Study 2 found that exposure to feminist conspiracy theories was associated with rape myths via increased belief in feminist conspiracy theories. Critically, the effects in both studies were conditional on higher levels of hostile sexist attitudes. Thus, our work supports the notion that conspiracy beliefs must align with underlying political predispositions (Douglas et al., 2019) and provides novel evidence that this includes sexist ideology. The links between feminist conspiracy beliefs and rape myths likely result from such a link upholding a person's sexist predispositions. Exploring avenues to address intergroup conspiracy theories while accounting for political ideologies is timely for researchers to consider in the future.

## Supplemental Material

sj-docx-1-vaw-10.1177_10778012241234892 - Supplemental material for Sexism and Feminist Conspiracy Beliefs: Hostile Sexism Moderates the Link Between Feminist Conspiracy Beliefs and Rape Myth Acceptance Supplemental material, sj-docx-1-vaw-10.1177_10778012241234892 for Sexism and Feminist Conspiracy Beliefs: Hostile Sexism Moderates the Link Between Feminist Conspiracy Beliefs and Rape Myth Acceptance by Daniel Jolley, Silvia Mari, Tanya Schrader and Darel Cookson in Violence Against Women

## References

[bibr1-10778012241234892] Abrams D. Viki G. T. Masser B. Bohner G. (2003). Perceptions of stranger and acquaintance rape: The role of benevolent and hostile sexism in victim blame and rape proclivity. Journal of Personality and Social Psychology, 84(1), 111–125. 10.1037/0022-3514.84.1.111 12518974

[bibr2-10778012241234892] Angelone D. J. Cantor N. Marcantonio T. Joppa M. (2021). Does sexism mediate the gender and rape myth acceptance relationship? Violence Against Women, 27(6–7), 748–765. 10.1177/1077801220913632 32339090

[bibr3-10778012241234892] Bilewicz M. Winiewski M. Kofta M. Wójcik A. (2013). Harmful ideas, the structure and consequences of anti-semitic beliefs in Poland. Political Psychology, 34(6), 821–839. 10.1111/pops.12024

[bibr4-10778012241234892] Brotherton R. French C. C. Pickering A. D. (2013). Measuring belief in conspiracy theories: The generic conspiracist beliefs scale. Frontiers in Psychology, 4, 279. 10.3389/fpsyg.2013.00279 23734136 PMC3659314

[bibr5-10778012241234892] Burt M. R. (1980). Cultural myths and supports for rape. Journal of Personality and Social Psychology, 38(2), 217–230. 10.1037/0022-3514.38.2.217 7373511

[bibr6-10778012241234892] Claassen R. L. Ensley M. J. (2016). Motivated reasoning and yard-sign-stealing partisans: Mine is a likable rogue, yours is a degenerate criminal. Political Behavior, 38(2), 317–335. 10.1007/s11109-015-9313-9

[bibr7-10778012241234892] Connor R. A. Fiske S. T. (2018). Warmth and competence: A feminist look at power and negotiation. In Travis C. B. White J. W. Rutherford A. Williams W. S. Cook S. L. Wyche K. F. (Eds.), APA handbook of the psychology of women: History, theory, and battlegrounds (pp. 321–342). American Psychological Association.

[bibr8-10778012241234892] Davies M. Gilston J. Rogers P. (2012). Examining the relationship between male rape myth acceptance, female rape myth acceptance, victim blame, homophobia, gender roles, and ambivalent sexism. Journal of Interpersonal Violence, 27(14), 2807–2823. 10.1177/0886260512438281 22550150

[bibr9-10778012241234892] Douglas K. M. Uscinski J. E. Sutton R. M. Cichocka A. Nefes T. Ang C. S. Deravi F. (2019). Understanding conspiracy theories. Political Psychology, 40(S1), 3–35. 10.1111/pops.12568

[bibr10-10778012241234892] Egan R. Wilson J. C. (2012). Rape victims’ attitudes to rape myth acceptance. Psychiatry, Psychology and Law, 19(3), 345–357. 10.1080/13218719.2011.585128

[bibr11-10778012241234892] Fiske S. T. Cuddy A. J. C. Glick P. Xu J. (2002). A model of (often mixed) stereotype content: Competence and warmth respectively follow from perceived status and competition. Journal of Personality and Social Psychology, 82(6), 878–902. 10.1037/0022-3514.82.6.878 12051578

[bibr12-10778012241234892] Glick P. Fiske S. T. (1996). The ambivalent sexism inventory: Differentiating hostile and benevolent sexism. Journal of Personality and Social Psychology, 70(3), 491–512. 10.1037/0022-3514.70.3.491

[bibr13-10778012241234892] Glick P. Fiske S. T. (1997). Hostile and benevolent sexism. Psychology of Women Quarterly, 21(1), 119–135. 10.1111/j.1471-6402.1997.tb00104.x PMC3896071 24453402

[bibr14-10778012241234892] Gualda E. Castillo Algarra J. González Gómez T. Morales Marente E. Palacios Gálvez M. S. Rodríguez Pascual I., Rúas Araujo J. (2019). *Conspiracy theories and disinformation in Andalusia* (Executive Report 2019). https://rabida.uhu.es/dspace/bitstream/handle/10272/16291/Conspiracy%20Theories%20Disinformation%20in%20Andalusia_ExecutiveReport%202019.pdf?sequence=2

[bibr15-10778012241234892] Hayes, A. F. (2013). *Introduction to mediation, moderation, and conditional process analysis: A regression-based approach*. Guildford Press.

[bibr16-10778012241234892] Hayes A. F. (2015). An index and test of linear moderated mediation. Multivariate Behavioral Research, 50(1), 1–22. 10.1080/00273171.2014.962683 26609740

[bibr17-10778012241234892] Hayes A. F. Matthes J. (2009). Computational procedures for probing interactions in OLS and logistic regression: SPSS and SAS implementations. Behavior Research Methods, 41(3), 924–936. 10.3758/BRM.41.3.924 19587209

[bibr18-10778012241234892] Hayes A. F. Rockwood N. J. (2017). Regression-based statistical mediation and moderation analysis in clinical research: Observations, recommendations, and implementation. Behaviour Research and Therapy, 98, 39–57. 10.1016/j.brat.2016.11.001 27865431

[bibr19-10778012241234892] Hopton K. Langer S. (2022). Kick the XX out of your life: An analysis of the manosphere's discursive constructions of gender on Twitter. Feminism & Psychology, 32(1), 3–22. 10.1177/09593535211033461

[bibr20-10778012241234892] Hornsey M. J. Pearson S. (2022). Cross-national differences in willingness to believe conspiracy theories. Current Opinion in Psychology, 47, 101391. 10.1016/j.copsyc.2022.101391 35830765

[bibr21-10778012241234892] Jolley D. Douglas K. M. (2014). The effects of anti-vaccine conspiracy theories on vaccination intentions. PLoS ONE, 9(2), e89177. 10.1371/journal.pone.0089177 PMC3930676 24586574

[bibr22-10778012241234892] Jolley D. Marques M. D. Cookson D. (2022). Shining a spotlight on the dangerous consequences of conspiracy theories. Current Opinion in Psychology, 47, 101363. 10.1016/j.copsyc.2022.101363 35732091 PMC9142208

[bibr23-10778012241234892] Jolley D. Meleady R. Douglas K. M. (2020). Exposure to intergroup conspiracy theories promotes prejudice which spreads across groups. British Journal of Psychology, 111(1), 17–35. 10.1111/bjop.12385 30868563 PMC7004178

[bibr24-10778012241234892] Kempker E. M. (2018). Big sister: Feminism, conservatism, and conspiracy in the Heartland. University of Illinois Press.

[bibr25-10778012241234892] Krahé B. Temkin J. Bieneck S. Berger A. (2008). Prospective lawyers’ rape stereotypes and schematic decision making about rape cases. Psychology, Crime & Law, 14(5), 461–479. 10.1080/10683160801932380

[bibr26-10778012241234892] Kunst J. R. Bailey A. Prendergast C. Gundersen A. (2019). Sexism, rape myths and feminist identification explain gender differences in attitudes toward the #metoo social media campaign in two countries. Media Psychology, 22(5), 818–843. 10.1080/15213269.2018.1532300

[bibr27-10778012241234892] Lerner M. J. (1997). What does the belief in a just world protect US from: The dread of death or the fear of understanding suffering? Psychological Inquiry, 8(1), 29–32. https://doi.org/https://doi/10.1207/s15327965pli0801_5

[bibr28-10778012241234892] Lonsway K. A. Fitzgerald L. F. (1994). Rape myths. Psychology of Women Quarterly, 18(2), 133–164. 10.1111/j.1471-6402.1994.tb00448.x

[bibr29-10778012241234892] Marchlewska M. Cichocka A. Łozowski F. Górska P. Winiewski M. (2019). In search of an imaginary enemy: Catholic collective narcissism and the endorsement of gender conspiracy beliefs. The Journal of Social Psychology, 159(6), 766–779. 10.1080/00224545.2019.1586637 30870100

[bibr30-10778012241234892] Marques J. M. Paez D. (1994). The ‘black sheep effect’: Social categorization, rejection of ingroup deviates, and perception of group variability. European Review of Social Psychology, 5(1), 37–68. 10.1080/14792779543000011

[bibr31-10778012241234892] Marwick A. E. Caplan R. (2018). Drinking male tears: Language, the manosphere, and networked harassment. Feminist Media Studies, 18, 543–559. 10.1080/14680777.2018.1450568

[bibr32-10778012241234892] Maxwell L. Scott G. (2014). A review of the role of radical feminist theories in the understanding of rape myth acceptance. Journal of Sexual Aggression, 20(1), 40–54. 10.1080/13552600.2013.773384

[bibr33-10778012241234892] McMahon S. Farmer G. L. (2011). An updated measure for assessing subtle rape myths. Social Work Research, 35(2), 71–81. 10.1093/swr/35.2.71

[bibr34-10778012241234892] O'Connor J. (2021). The longitudinal effects of rape myth beliefs and rape proclivity. Psychology of Men & Masculinities, 22(2), 321. https://doi.org/10.1037%2Fmen0000324 35979223 10.1037/men0000324 PMC9380434

[bibr35-10778012241234892] Payne D. L. Lonsway K. A. Fitzgerald L. F. (1999). Rape myth acceptance: Exploration of its structure and its measurement using the Illinois rape myth acceptance scale. Journal of Research in Personality, 33(1), 27–68. 10.1006/jrpe.1998.2238

[bibr36-10778012241234892] Ramos M. Barreto M. Ellemers N. Moya M. Ferreira L. (2018). What hostile and benevolent sexism communicate about men's and women's warmth and competence. Group Processes & Intergroup Relations, 21(1), 159–177. 10.1177/1368430216656921

[bibr37-10778012241234892] Ratliff K. A. Nosek B. A. (2011). Negativity and outgroup biases in attitude formation and transfer. Personality and Social Psychology Bulletin, 37(12), 1692–1703. 10.1177/0146167211420168 21885857

[bibr38-10778012241234892] Robnett R. D. Anderson K. J. Hunter L. E. (2012). Predicting feminist identity: Associations between gender-traditional attitudes, feminist stereotyping, and ethnicity. Sex Roles, 67(3–4), 143–157. 10.1007/s11199-012-0170-2

[bibr39-10778012241234892] Suarez E. Gadalla T. M. (2010). Stop blaming the victim: A meta-analysis on rape myths. Journal of Interpersonal Violence, 25(11), 2010–2035. 10.1177/0886260509354503 20065313

[bibr40-10778012241234892] van Prooijen J.‐W. Douglas K. M. (2017). Conspiracy theories as part of history: The role of societal crisis situations. Memory Studies, 10(3), 323–333. 10.1177/1750698017701615 29081831 PMC5646574

[bibr41-10778012241234892] Young Women's Trust. (2019). *Young women's feminism and activism 2019*. https://www.youngwomenstrust.org/assets/0001/2668/Activism_2019.pdf

