# Supplementary material for: Sexism and Feminist Conspiracy Beliefs: Hostile Sexism Moderates the Link Between Feminist Conspiracy Beliefs and Rape Myth Acceptance
Source: Violence Against Women. 2024 Mar 4;31(6-7):1447–68. doi: 10.1177/10778012241234892 (PMC11969872; doi:10.1177/10778012241234892)
Supplement: sj-docx-1-vaw-10.1177_10778012241234892 - Supplemental material for Sexism and Feminist Conspiracy Beliefs: Hostile Sexism Moderates the Link Between Feminist Conspiracy Beliefs and Rape Myth Acceptance [file sj-docx-1-vaw-10.1177_10778012241234892.docx]

**Sexism and feminist conspiracy beliefs: Hostile sexism moderates the link between feminist conspiracy beliefs and rape myth acceptance**

**Supplementary Materials**

**Factor structure of hostile sexism and belief in feminist conspiracy theories**

As feminist conspiracy theories and hostile sexism both present women (in general) in a negative light, an exploratory factor analysis (EFA) was performed to examine the factor structure of the measures. In Study 1, an EFA was conducted using oblique orthogonal (Varimax) to allow simple identification on whether the one feminist conspiracy theory item loaded on the hostile sexism measure (11 items). Statistical assumptions were met and the analysis revealed two factors with eigenvalues > 1, explaining 46.55 per cent and 10.82 per cent of the variance respectively. The two factors comprised of the hostile sexism items, where the negative items cross-loaded on to the second factor - however, the results reported in the main text were not affected when the negative items were omitted. As expected, the feminist conspiracy belief item did not load substantially on either factor. This provides evidence that hostile sexism and conspiracy beliefs are separate constructs.

As in Study 1, an EFA was run on the individual items of feminist conspiracy theories (5 items) and hostile sexism (11 items) scales in Study 2 using oblique rotation (Promax). Statistical assumptions were met and the analysis revealed three factors with eigenvalues > 1, explaining 58.47 per cent, 8.25 per cent and 6.35 per cent of the variance respectively. As in Study 1, two factors comprised of the hostile sexism items, where the negative items cross-loaded on to another factor - however, the results reported in the main text were not affected when the negative items were omitted. The conspiracy items loaded substantially on the predicted scale. The EFA provides further confidence that the measure of belief in feminist conspiracy theories and hostile sexism are separate constructs.

**Gender differences between each of the key measures in Study 1**

*Table S1.*

Welch's t-test examining the difference between gender on key variables in Study 1 (n = 199).

| **Variable Explained** | **Gender (M [*SD*])** | | **Welch's t-test** | | | |
| --- | --- | --- | --- | --- | --- | --- |
|  | Male (*n* = 53) | Female (*n* = 146) | *t* | *df* | *p* | *d* |
| Feminist identification | 4.28 *(1.28)* | 4.83 *(1.44)* | -2.433 | 103.234 | .011 | 0.40 |
| Feminist conspiracy | 2.53 (*1.42*) | 2.16 (*1.19*) | 1.695 | 79.937 | .094 | 0.28 |
| General conspiracy | 3.91 (*1.38*) | 3.85 (*1.35*) | 0.266 | 90.259 | .791 | 0.04 |
| Benevolent sexism | 3.33 (*1.06*) | 2.87 (*0.92*) | 2.808 | 82.681 | .006 | 0.46 |
| Hostile sexism | 3.07 (*1.18*) | 2.73 (*1.12*) | 1.850 | 88.589 | .068 | 0.30 |
| Belief in a just world | 3.56 (*1.09*) | 3.62 (*1.21*) | -0.311 | 101.553 | .756 | 0.05 |
| Rape myth acceptance | 2.16 (*0.84*) | 1.73 (*0.69*) | 3.293 | 79.082 | .001 | 0.56 |

**Means and Pearson product-moment correlations in Study 2**

*Table S2.*

Means and Pearson product-moment correlations for all variables in Study 2 across experimental conditions (n = 578).

|  |  | M  (*SD*) | 1 | 2 | 3 | 4 | 5 | 6 |
| --- | --- | --- | --- | --- | --- | --- | --- | --- |
| 1 | Age | 40.20 (12.39) | - | -.09* | .20*** | .06 | .03 | .13* |
| 2 | Feminist identity | 4.44 (1.27) |  | - | -.51*** | -.58*** | -.60*** | -.46*** |
| 3 | Political orientation | 3.28 (1.41) |  |  | - | .44*** | .45*** | .41*** |
| 4 | Feminist conspiracy beliefs | 2.46 (*1.44*) |  |  |  | - | .73*** | .59*** |
| 5 | Hostile sexism | 2.93 (*1.04*) |  |  |  |  | - | .68*** |
| 6 | Rape myth acceptance | 1.94 (*0.81*) |  |  |  |  |  | - |

*Notes*. **p* < .05. ***p* <. 01. ****p* <. 001.

**Gender differences between each of the key measures in Study 2**

*Table S3.*

T-test examining the difference between gender on key variables in Study 2 (n = 570)

| **Variable Explained** | **Gender (M [*SD*])** | | **T-test** | | | |
| --- | --- | --- | --- | --- | --- | --- |
|  | Male (*n* = 288) | Female (*n* = 282) | *t* | *df* | *p* | *d* |
| Feminist identification | 4.29 (*1.24)* | 4.57 *(1.26)* | -0.699 | 568 | .004 | 0.23 |
| Feminist conspiracy beliefs | 2.68 (1.47) | 2.25 (1.39) | 3.633 | 568 | <.001 | 0.30 |
| Hostile sexism | 3.21 (1.16) | 2.66 (0.88) | 6.527 | 567.168 | <.001 | 0.55 |
| Rape myth acceptance | 2.11 (0.85) | 1.76 (0.72) | 5.317 | 555.238 | <.001 | 0.45 |
